# EPIGENETIC AND METABOLIC REGULATION OF AGING

**DOI:** 10.1093/geroni/igz038.871

**Published:** 2019-11-08

**Authors:** Anne Brunet

**Affiliations:** Stanford University, Stanford, California, United States

## Abstract

Aging is accompanied by a decline in the regenerative potential of most tissues. The mammalian brain contains regenerative neurogenic niches composed of neural stem cells (NSCs), neural progenitors, and other cells, including microglia, and endothelial cells. Neurogenic niches become less functional with increasing age. This deterioration could underlie cognitive and sensory restriction with age, although the exact age at which it occurs is still debated in humans. How the neurogenic niche changes during aging, and whether new cell types arise in older individuals, is not known. Our lab has embarked on a global characterization of the neurogenic niche during aging. This work provides a global understanding of the old neurogenic niche and suggests possible cause for NSC decline during aging. Results from these studies could open new avenues to counter age-related decline in the neurogenic niche and brain aging.

